# Which immunonutritional marker best predicts disease-free survival in non-metastatic colorectal Cancer? The CALLY index and CAR emerge as independent predictors from a seven-marker comparison

**DOI:** 10.3389/fnut.2026.1865242

**Published:** 2026-06-18

**Authors:** Jong Min Lee, Taehyung Kim, Nam Kyu Kim

**Affiliations:** Department of Surgery, Yongin Severance Hospital, Yonsei University College of Medicine, Yongin, Republic of Korea

**Keywords:** colorectal neoplasms, C-reactive protein, disease-free survival, lymphocyte count, nutritional status, prognosis, serum albumin

## Abstract

**Background:**

Many immunonutritional markers (INMs) have emerged as significant prognostic indicators in colorectal cancer (CRC); however, limited attention has been given to directly comparing these candidates to identify the most informative predictors.

**Methods:**

We retrospectively analyzed 414 patients with stage I–III CRC who underwent curative resection. Seven INMs, including the C-reactive protein-albumin-lymphocyte (CALLY) index, the C-reactive protein-to-albumin ratio (CAR), and five other markers, were evaluated. The primary outcome was disease-free survival (DFS). Multivariable Cox proportional hazards regression was performed to identify independent predictors of DFS. The discriminatory performance of the resulting models was quantified using Harrell’s concordance index (C-index).

**Results:**

In univariable analysis, all seven INMs were significantly associated with DFS. However, after adjusting for T and N stages and other covariates, only the CALLY index (HR, 1.686; 95% confidence interval (CI), 1.038–2.738; *p* = 0.035) and CAR (HR, 2.089; 95% CI, 1.276–3.422; *p* = 0.004) remained independent predictors. Integrating these markers into the staging system significantly enhanced predictive accuracy. The C-index improved from 0.713 (95% CI, 0.657–0.764) for the stage-only model to 0.812 (95% CI, 0.781–0.867) for the CALLY + stage model and 0.809 (95% CI, 0.779–0.865) for the CAR + stage model.

**Conclusion:**

The CALLY index and CAR were the only INMs that retained independent prognostic significance after adjustment for T and N stages. Incorporating these continuous markers into the Tumor-Node-Metastasis (TNM) staging system substantially improved risk stratification and predictive accuracy in patients with non-metastatic CRC.

## Introduction

Colorectal cancer (CRC) ranks as the third most frequently diagnosed cancer and the second leading cause of cancer-related mortality worldwide (1). The Tumor-Node-Metastasis (TNM) staging system remains the gold standard for predicting prognosis in CRC; however, it is primarily based on the anatomical extent of the tumor and does not incorporate host-related biological factors such as the systemic inflammatory response or nutritional status (2).

Systemic inflammatory responses and nutritional status are increasingly recognized as critical prognostic indicators in various malignancies (3). While initial research focused primarily on the relationship between systemic inflammation and prognosis, more recent studies have emphasized indices that incorporate serum albumin as a reflection of both nutritional reserve and chronic inflammation (4, 5) Among these, the prognostic nutritional index (PNI) and the modified Glasgow Prognostic Score (mGPS) have been studied most extensively, often providing superior prognostic impact by accounting for the host’s nutritional status (2, 3).

While nutritional depletion has long been associated with short-term surgical morbidity, its impact on long-term oncological outcomes is increasingly recognized through immunological mechanisms. Protein-energy malnutrition compromises cell-mediated immune responses, including T-cell proliferation, natural killer cell cytotoxicity, and pro-inflammatory cytokine production, thereby weakening immunosurveillance against residual tumor cells after curative resection (6). At the same time, tumor-driven systemic inflammation accelerates protein catabolism and depletes nutritional reserves, creating a vicious cycle in which malnutrition and immune dysfunction reinforce each other (7). Accordingly, immunonutritional markers (INMs) constructed from inflammatory and nutritional parameters may capture this bidirectional interaction.

Despite the proliferation of various INMs, these indices have not been widely incorporated into routine clinical practice. A possible contributing factor is that the existing literature has largely focused on validating individual, preselected indices in isolation, with relatively limited effort devoted to comparative evaluation among competing markers (8–10). Moreover, many of the proposed indices are constructed from a similar set of readily available laboratory parameters, raising the question of whether incremental modifications in calculation formulas yield meaningfully distinct prognostic information. A systematic comparison across multiple candidate markers may therefore help clarify which indices offer genuinely independent predictive value.

Furthermore, evaluations of independent prognostic value in the existing literature often rely on models adjusted only for overall TNM stage (I–IV) (11–15). However, as demonstrated by the “survival paradox”—in which patients with T4N0 (Stage II) can exhibit poorer outcomes than those with T1-2N1 (Stage IIIA)—adjusting only for overall stage may obscure the specific impact of tumor invasiveness and nodal involvement (16). Adjusting for T and N stages separately could provide a reliable evaluation of the prognostic significance of INMs.

Therefore, we aimed to identify preoperative INMs that serve as independent predictors of survival in patients with nonmetastatic CRC and to determine whether incorporating these indices would provide incremental prognostic value beyond that of the TNM staging system.

## Materials and methods

### Study design and patients

We retrospectively reviewed a prospectively maintained database of patients who underwent surgical resection for colorectal tumors at Yongin Severance Hospital between March 2020 and December 2022. Initially, 568 patients who underwent resection during this period were identified. Patients were excluded based on the following criteria: histologically confirmed neuroendocrine or gastrointestinal stromal tumors (*n* = 4); anal squamous cell carcinoma or melanoma (*n* = 7); colonic adenoma (*n* = 3); stage IV CRC (*n* = 116); recurrent CRC (*n* = 2); or palliative resection (*n* = 22). After applying these exclusion criteria, a total of 414 patients with nonmetastatic colorectal adenocarcinoma who underwent curative-intent surgery were included in the final analysis (Supplementary Figure S1). This study conformed to the Strengthening of the Reporting of Observational Studies in Epidemiology (STROBE) guidelines.

### Data collection and variables

Clinical and pathological data associated with patient prognosis were collected. Patient-related variables included demographics (age, sex, and body mass index [BMI]) and American Society of Anesthesiologists physical status (ASA PS). Tumor and treatment-related variables comprised tumor location (right colon, left colon, or rectum), pathological TNM classification, tumor (T) and node (N) stages, carcinoembryonic antigen (CEA) level, histologic grade, lymphovascular invasion (LVI), perineural invasion (NI), R1 resection status, preoperative treatment (radiotherapy or chemotherapy), and adjuvant chemotherapy (CTx).

Preoperative laboratory data required to calculate various INMs were retrieved. These markers included the advanced lung cancer inflammation index (ALI) (15, 17), the hemoglobin, albumin, lymphocyte, and platelet (HALP) score (18), the CRP-albumin-lymphocyte (CALLY) index (9, 11, 14), the C-reactive protein-to-albumin ratio (CAR) (5, 12), the controlling nutritional status (CONUT) score (19), PNI (13, 15), and mGPS (8, 10). All laboratory values were obtained from the most recent blood tests performed within 2 months before surgery in accordance with the institutional anesthetic protocol. The calculation formulas and scoring systems for all INMs are provided in Table 1.

**Table 1:** Definitions and calculation formulas of the seven immuno-nutritional markers.

Marker	Calculation formula/scoring system
ALI	BMI × Alb (g/dL) × Lym (10^3^/μL)/Neu (10^3^/μL)
HALP	[Hb (g/dL) × Alb (g/dL) × Lym (10^3^/μL)]/Plt (10^3^/μL)
CALLY	[Alb (g/dL) × Lym (10^3^/μL)]/[CRP (mg/dL) × 10]
CAR	CRP (mg/dL)/Alb (g/dL)
PNI	10 × Alb (g/dL) + 5 × Lym (10^3^/μL)
CONUT*	Sum of scores from serum Alb, total Lym count, and total cholesterol levels (Score range: 0–12)
mGPS^†^	Based on CRP (> 1.0 mg/dL) and Alb (< 3.5 g/dL) levels (Score: 0, 1, or 2)

*CONUT scoring system: the total score (0–12) is calculated by summing the scores from three components.

Alb (g/dL): 3.5 (0 points); 3.0–3.49 (2 points); 2.5–2.99 (4 points); < 2.5 (6 points).

Lym (10^3^/μL): 1.6 (0 points); 1.2–1.59 (1 point); 0.8–1.19 (2 points); < 0.8 (3points).

Total cholesterol (mg/dL): 180 (0 points); 140–179 (1 point); 100–139 (2 points); < 100 (3 points).

^†^mGPS scoring system.

Score 0: CRP ≤ 1.0 mg/dL.

Score 1: CRP > 1.0 mg/dL and Alb ≥ 3.5 g/dL.

Score 2: CRP > 1.0 mg/dL and Alb < 3.5 g/dL.

BMI, body mass index; Hb, hemoglobin; Alb, albumin; Lym, total lymphocyte count; Neu, absolute neutrophil count; Plt, platelet count; CRP, C-reactive protein; ALI, advanced lung cancer inflammation index; HALP, hemoglobin, albumin, lymphocyte, and platelet score; CALLY, CRP-albumin-lymphocyte index; CAR, C-reactive protein-to-albumin ratio; PNI, prognostic nutritional index; CONUT, controlling nutritional status score; mGPS, modified Glasgow Prognostic Score.

### Study outcome and surveillance

The primary outcome of this study was disease-free survival (DFS), defined as the interval from surgery to the first documentation of tumor recurrence or death from any cause. Patients who were alive and recurrence-free at the time of the last follow-up were censored.

Postoperative surveillance followed the institutional protocol, which typically included physical examinations, CEA measurements, and chest/abdominopelvic computed tomography (CT) scans every 3–6 months for the first 2 years and every 6–12 months thereafter. Recurrence was confirmed through histological biopsy or clear radiological evidence of new lesions.

### Statistical analyses

Continuous variables were presented as mean ± standard deviation or median with interquartile range (IQR), as appropriate, and categorical variables were expressed as frequencies. Missing values in predictive variables were negligible (<2.7%) and were addressed using multiple imputation by chained equations (MICE). Twenty imputed datasets were generated, and results were pooled according to Rubin’s rules.

For the primary outcome analysis, continuous INMs were categorized using optimal cutoff values determined by maximally selected rank statistics to maximize the prognostic difference in DFS. Survival curves were estimated using the Kaplan–Meier (KM) method and compared with the log-rank test. Univariable and multivariable Cox proportional hazards regression analyses were performed to identify independent prognostic factors, with results reported as hazard ratios (HRs) and 95% confidence intervals (CIs). Because the seven INMs share overlapping components, each was assessed in a separate multivariable model together with the same covariates to avoid multicollinearity.

Model discriminatory performance was evaluated using Harrell’s concordance index (C-index). Bootstrapping with 1,000 resamples was employed to estimate the 95% CIs for the C-index and to calculate *p*-values for pairwise comparisons between the stage-only and integrated models to assess the incremental predictive value of the markers. All statistical analyses were performed using R software (version 4.5.1; R Foundation for Statistical Computing, Vienna, Austria). A two-sided *p*-value < 0.05was considered statistically significant.

## Results

### Patient characteristics and Immunonutritional marker categorization

The median follow-up for the included patients (*N* = 414) was 36.3 months (IQR, 19.6–44.1 months). For disease-free survivors, the median follow-up was 39.0 months. The prognostic performance of all INMs was evaluated. As shown in Supplementary Table S1, all markers exhibited significant predictive potential for DFS, with C-indices ranging from 0.560 to 0.663. Among them, the PNI showed the highest predictive accuracy (C-index, 0.663; 95% CI, 0.603–0.723), followed by ALI (0.651) and the CALLY index (0.649).

The optimal cut-off values for individual INMs were as follows: ALI (25.84), HALP (0.37), CALLY (1.43), CAR (0.13), and PNI (53.35). Patients were stratified into high-risk groups associated with poor survival: 123 (29.7%) had low ALI, 218 (52.7%) had low HALP, 129 (31.2%) had low CALLY, 119 (28.7%) had high CAR, and 247 (59.7%) had low PNI. For score-based nutritional indices, the CONUT score was categorized into three levels: normal (≤ 1), light (2–4), and moderate-to-severe (> 4). The mGPS was classified based on its standard clinical definition. Detailed clinicopathological and immunonutritional characteristics of the included patients are summarized in Table 2.

**Table 2:** Baseline clinic pathological and immunonutritional characteristics of the study population.

Variables	*N* = 414
Age >70 years	155 (37.4)
Male sex	209 (50.5)
BMI > 25 kg/m^2^	164 (39.6)
ASA PS ≥ III	123 (29.7)
CEA > 5 ng/mL	149 (36.0)
Tumor location	
Left colon	119 (29.7)
Right colon	125 (28.7)
Rectum	170 (41.1)
Neoadjuvant Tx.	57 (13.8)
T stage	
1–2	120 (29.0)
3	243 (58.7)
4	51 (12.3)
N stage	
0	238 (57.5)
1	115 (27.8)
2	61 (14.7)
TNM stage	
1	99 (23.9)
2	139 (33.6)
3	176 (42.5)
PD or Muc or SRC	34 (8.2)
LVI	223 (53.9)
NI	140 (33.8)
R1 resection	31 (7.5)
Adjuvant CTx	237 (68.8)
Immuno-nutritional markers	
ALI, median (IQR)	44.79 (24.33–66.48)
HALP, median (IQR)	0.36 (0.21–0.53)
CALLY, median (IQR)	3.83 (1.00–10.19)
CAR, median (IQR)	0.04 (0.01–0.17)
PNI, median (IQR)	51.25 (46.51–55.84)
CONUT	
≤1	197 (47.6)
2–4	183 (44.2)
>4	34 (8.2)
mGPS	
0	339 (81.9)
1	49 (11.8)
2	26 (6.3)

Data are presented as *n* (%) for categorical variables and median (interquartile range) for continuous variables. The reported values are derived from the first representative dataset among the multiple imputations.

ASA PS, American Society of Anesthesiologists Physical Status; BMI, body mass index; CEA, carcinoembryonic antigen; T, tumor; N, node; TNM, tumor-node-metastasis; PD, poorly differentiated adenocarcinoma; Muc, mucinous adenocarcinoma; SRC, signet ring cell carcinoma; LVI, lymphovascular invasion; NI, neural invasion; Adjuvant CTx, adjuvant chemotherapy; ALI, advanced lung cancer inflammation index; HALP, hemoglobin, albumin, lymphocyte, and platelet score; CALLY, CRP-albumin-lymphocyte index; CAR, C-reactive protein-to-albumin ratio; PNI, prognostic nutritional index; CONUT, controlling nutritional status score; mGPS, modified glasgow prognostic score.

### Univariable analysis

In the univariable analysis, older age (>70 years), elevated preoperative CEA levels (>5 ng/mL), and neoadjuvant therapy were significant risk factors for DFS. Advanced TNM stage, T and N stages, LVI, PNI, and R1 resection were significantly associated with worse survival outcomes. All immunonutritional groups assessed (ALI, HALP, CALLY, CAR, PNI, CONUT, and mGPS) were significantly associated with DFS (Table 3).

**Table 3:** Univariable cox proportional hazards models for factors associated with disease-free survival.

Variables	Univariable	
	HR (95% CI)	*p*
Age >70 years	2.108 (1.294–3.622)	0.003
Male sex	0.940 (0.602–1.468)	0.781
BMI > 25 kg/m^2^	0.802 (0.504–1.276)	0.347
ASA PS ≥ III	1.419 (0.880–2.289)	0.149
CEA > 5 ng/ml	3.354 (2.101–5.356)	< 0.001
Tumor location		
Left colon	Ref	
Right colon	0.785 (0.414–1.489)	0.453
Rectum	1.350 (0.791–2.304)	0.267
Neoadjuvant Tx.	2.762 (1.675–4.576)	< 0.001
T stage		
1–2	Ref	
3	6.165 (2.479–15.334)	< 0.001
4	9.992 (3.667–27.225)	< 0.001
N stage		
0	Ref	
1	2.020 (1.155–3.533)	0.014
2	5.037 (2.951–8.599)	< 0.001
TNM stage		
I	Ref	
II	2.402 (1.001–5.760)	0.049
III	5.282 (2.370–11.772)	< 0.001
PD or Muc or SRC	1.081 (0.492–2.377)	0.844
LVI	1.672 (1.038–2.693)	0.035
NI	2.899 (1.836–4.576)	< 0.001
R1 resection	2.719 (1.455–5.081)	0.002
Adjuvant CTx	2.165 (1.294–3.622)	0.003
Immuno-nutritional markers		
ALI < 25.84	2.798 (1.784–4.387)	< 0.001
HALP < 0.37	1.920 (1.205–3.411)	0.004
CALLY < 1.43	2.386 (1.520–3.745)	< 0.001
CAR > 0.13	2.490 (1.584–3.915)	< 0.001
PNI < 53.35	3.222 (1.867–5.561)	< 0.001
CONUT		
≤1	Ref	
2–4	2.082 (1.271–3.411)	0.004
>4	4.345 (2.052–9.199)	< 0.001
mGPS		
0	Ref	
1	2.102 (1.182–3.737)	0.012
2	0.937 (0.288–3.044)	0.912

HR, hazard ratio; CI, confidence interval; ASA PS, American Society of Anesthesiologists Physical Status; BMI, body mass index; CEA, carcinoembryonic antigen; T, tumor; N, node; TNM, tumor-node-metastasis; PD, poorly differentiated adenocarcinoma; Muc, mucinous adenocarcinoma; SRC, signet ring cell carcinoma; LVI, lymphovascular invasion; NI, neural invasion; R1, microscopic residual tumor; Adjuvant CTx, adjuvant chemotherapy; ALI, advanced lung cancer inflammation index; HALP, hemoglobin, albumin, lymphocyte, and platelet score; CALLY, CRP-albumin-lymphocyte index; CAR, C-reactive protein-to-albumin ratio; PNI, prognostic nutritional index; CONUT, controlling nutritional status score; mGPS, modified Glasgow Prognostic Score; Ref, reference category.

### Multivariable analysis

Multivariable Cox regression models were constructed for each immuno-nutritional group, adjusting for covariates identified in the univariable analysis. The proportional hazards assumption was satisfied for all variables in both models (global *p* = 0.309 and 0.361 for the CALLY and CAR models, respectively). In Model 1, adjusted for TNM stage, lower ALI, lower CALLY index, higher CAR, and lower PNI were independent predictors of DFS. In Model 2, which incorporated T and N stages instead of TNM stage, only low CALLY (< 1.43) (HR: 1.686; 95% CI: 1.038–2.738; *p* = 0.035) and high-CAR (> 0.13) (HR: 2.089; 95% CI: 1.276–3.422; *p* = 0.004) remained independent prognostic factors for DFS (Table 4).

**Table 4:** Multivariable cox proportional hazards models for each immunonutritional marker associated with disease-free survival.

Immunonutritional marker	Model 1 (TNM stage-adjusted)		Model 2 (T and N stage-adjusted)	
	HR (95% CI)	*p*	HR (95% CI)	*p*
ALI < 25.84	1.763 (1.062–2.926)	0.028	1.578 (0.960–2.593)	0.071
HALP < 0.37	1.106 (0.653–1.871)	0.704	1.021 (0.596–1.751)	0.938
CALLY < 1.43	1.847 (1.150–2.966)	0.011	1.686 (1.038–2.738)	0.035
CAR > 0.13	2.291 (1.413–3.716)	0.001	2.089 (1.276–3.422)	0.004
PNI < 53.35	1.985 (1.074–3.669)	0.029	1.758 (0.947–3.264)	0.073
CONUT				
≤ 1	Ref		Ref	
2–4	1.169 (0.678–2.016)	0.569	1.107 (0.639–1.918)	0.712
> 4	1.353 (0.580–3.153)	0.478	1.451 (0.627–3.357)	0.378
mGPS				
0	Ref		Ref	
1	1.645 (0.882–3.067)	0.115	1.608 (0.859–3.009)	0.135
2	0.629 (0.187–2.111)	0.447	0.444 (0.129–1.526)	0.193

Each row represents a separate multivariable analysis in which one specific immuno-nutritional marker was entered into the model along with the covariates listed below. Variables with *p* < 0.2 in the Univariable analysis were considered potential candidates for the multivariate models.

Model 1: adjusted for age (>70 years), ASA PS (≥III), preoperative CEA (>5 ng/ml), neoadjuvant Tx, TNM stage (I, II, or III), LVI, NI, R1 resection, and adjuvant CTx.

Model 2: adjusted for age (>70 years), ASA PS (≥III), preoperative CEA (>5 ng/mL), neoadjuvant Tx, T stage, N stage, LVI, NI, R1 resection, and adjuvant CTx.

HR, hazard ratio; CI, confidence interval; ASA PS, American Society of Anesthesiologists physical status; CEA, carcinoembryonic antigen; Tx, therapy; TNM, tumor-node-metastasis; LVI, lymphovascular invasion; NI, neural invasion; R1, microscopic residual tumor; CTx, chemotherapy; ALI, advanced lung cancer inflammation index; HALP, hemoglobin, albumin, lymphocyte, and platelet score; CALLY, CRP-albumin-lymphocyte index; CAR, C-reactive protein-to-albumin ratio; PNI, prognostic nutritional index; CONUT, controlling nutritional status score; mGPS, modified Glasgow Prognostic Score; Ref, reference category.

### Stage-stratified survival analysis using the CALLY index and CAR

**Figure 1:**
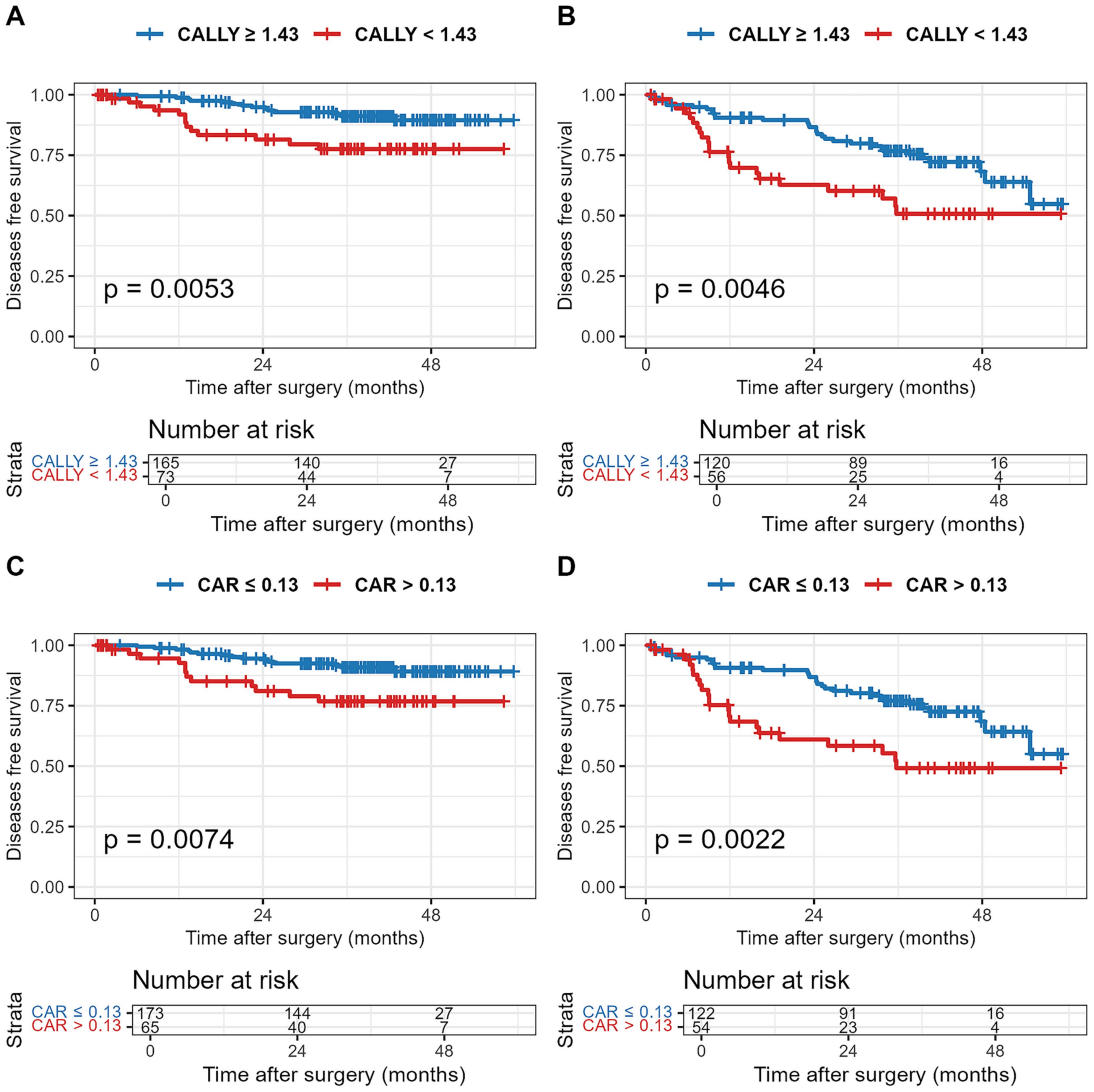
Kaplan–Meier survival curves for disease-free survival (DFS) according to the C-reactive protein-albumin-lymphocyte (CALLY) and C-reactive protein-to-albumin ratio (CAR)-group, stratified by stage. **(A)** CALLY index in stage I–II patients (3-year DFS rate: 91.1% vs. 77.5%, *p* = 0.005); **(B)** CALLY index in stage III patients (3-year DFS rate: 76.7% vs. 50.7%, *p* = 0.005); **(C)** CAR in stage I–II patients (3-year DFS rate: 90.9% vs. 76.8%, *p* = 0.007); and **(D)** CAR in stage III patients (3-year DFS rate: 77.1% vs. 49.2%, *p* = 0.002). DFS, disease-free survival; CAR, C-reactive protein-to-albumin ratio; CALLY, CRP-albumin-lymphocyte index.

Based on the multivariable analysis, in which only the CALLY index and CAR consistently demonstrated independent prognostic value in both Models 1 and 2, we further evaluated their clinical utility through stage-stratified survival analysis. For the CALLY index, patients in the low-CALLY group had significantly worse 3-year DFS than those in the normal-CALLY group in both stages I–II (77.5% vs. 91.1%; *p* = 0.005) and stage III (50.7% vs. 76.7%; *p* = 0.005). Similarly, high CAR was significantly associated with poorer survival than normal CAR in both stages I and II (3-year DFS rate: 76.8% vs. 90.9%; *p* = 0.007) and stage III (49.2% vs. 77.1%; *p* = 0.002) (Figure 1).

**Figure 2:**
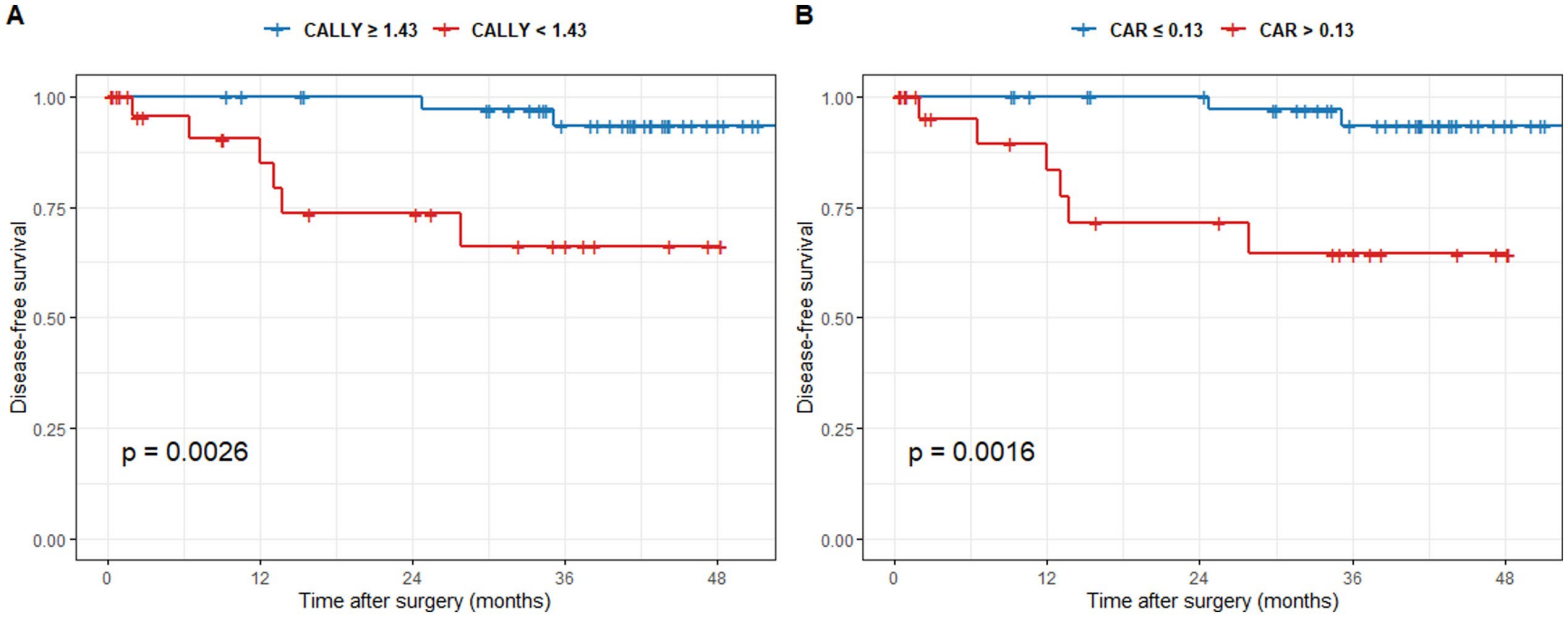
Kaplan–Meier survival curves for DFS according to the CALLY and CAR-group among stage II patients who did not receive adjuvant chemotherapy. **(A)** CALLY index (3-year DFS rate: 93.5% vs. 66.1%, *p* = 0.003); **(B)** CAR (3-year DFS rate: 93.5% vs. 64.4%, *p* = 0.002). DFS, disease-free survival; CALLY, CRP-albumin-lymphocyte index; CAR, C-reactive protein-to-albumin ratio.

To further explore the potential clinical utility of these markers in adjuvant treatment decision-making, we examined patients with stage II disease who did not receive adjuvant chemotherapy (*n* = 69)—a group generally considered to be at low risk. Even within this selected population, both low CALLY and high CAR identified patients with markedly worse outcomes. The 3-year DFS was 93.5% for the normal-CALLY group versus 66.1% for the low-CALLY group *(p* = 0.003), and 93.5% for the normal-CAR group versus 64.4% for the high-CAR group (*p* = 0.002) (Figure 2).

### Prognostic performance of the CALLY index and CAR in addition to pathological staging

**Figure 3:**
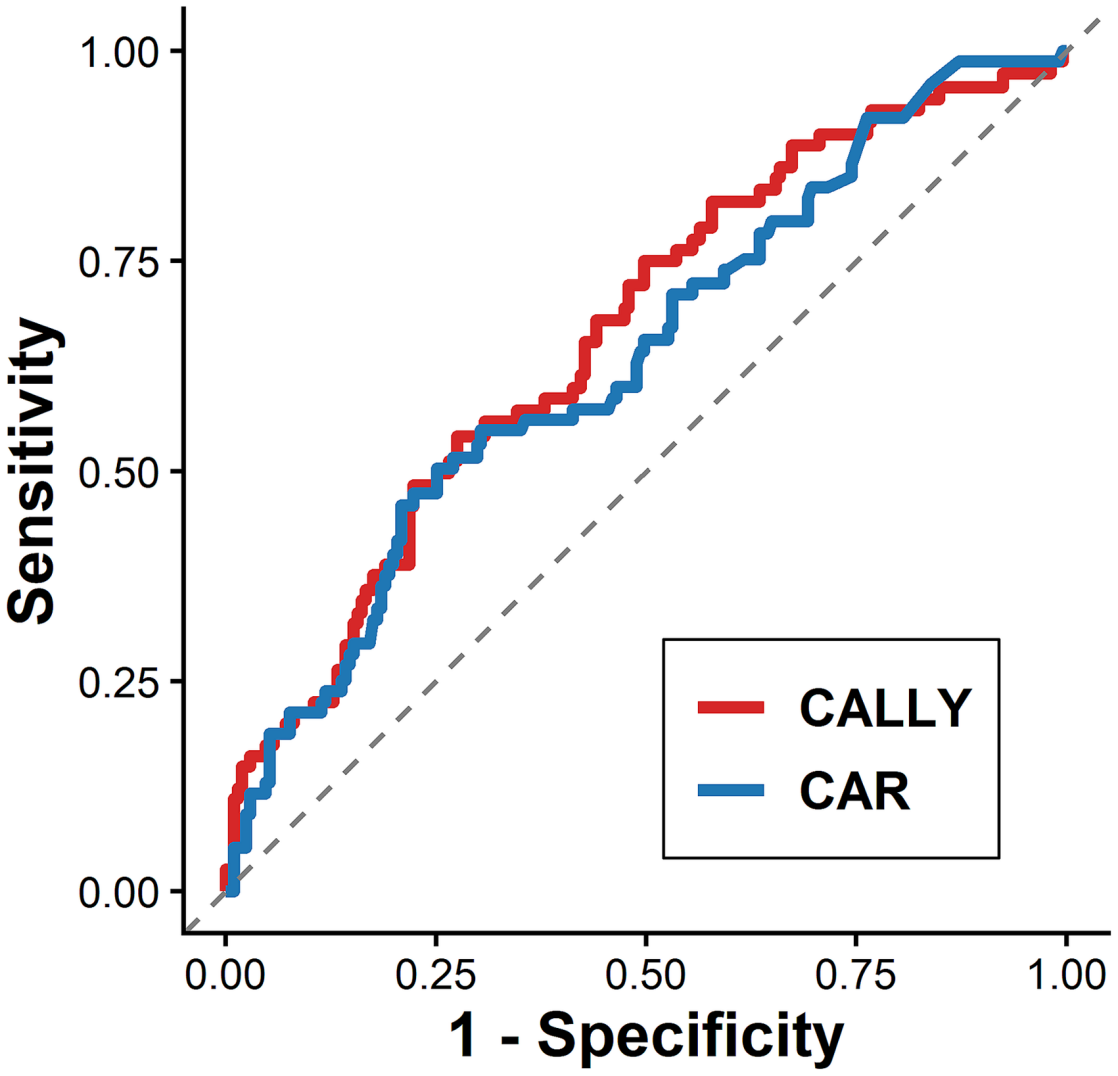
Time-dependent ROC curves of individual CAR and the CALLY index for predicting 3-year DFS. The AUC for 3-year DFS was 0.636 (95% CI, 0.560–0.708) for CAR and 0.663 (95% CI, 0.583–0.732) for the CALLY index. The observed difference in AUC was 0.027 (95% CI, −0.002 to 0.059; *p* = 0.079). AUC, area under the curve; CAR, C-reactive protein-to-albumin ratio; CALLY, CRP-albumin-lymphocyte index; CI, confidence interval; DFS, disease-free survival; ROC, receiver operating characteristic.

The overall predictive accuracy of the two INMs was evaluated using the C-index. Comparing individual performance, the CALLY index exhibited a slightly higher C-index than CAR (0.649; 95% CI, 0.582–0.705 vs. 0.622; 95% CI, 0.544–0.684), although this difference was not statistically significant (absolute difference, 0.027; 95% CI, −0.002 to 0.058; *p* = 0.224). Time-dependent ROC curve analysis for 3-year DFS also showed no significant difference in AUC between the CALLY index and CAR (0.663 vs. 0.636; *p* = 0.079; Figure 3).

**Figure 4:**
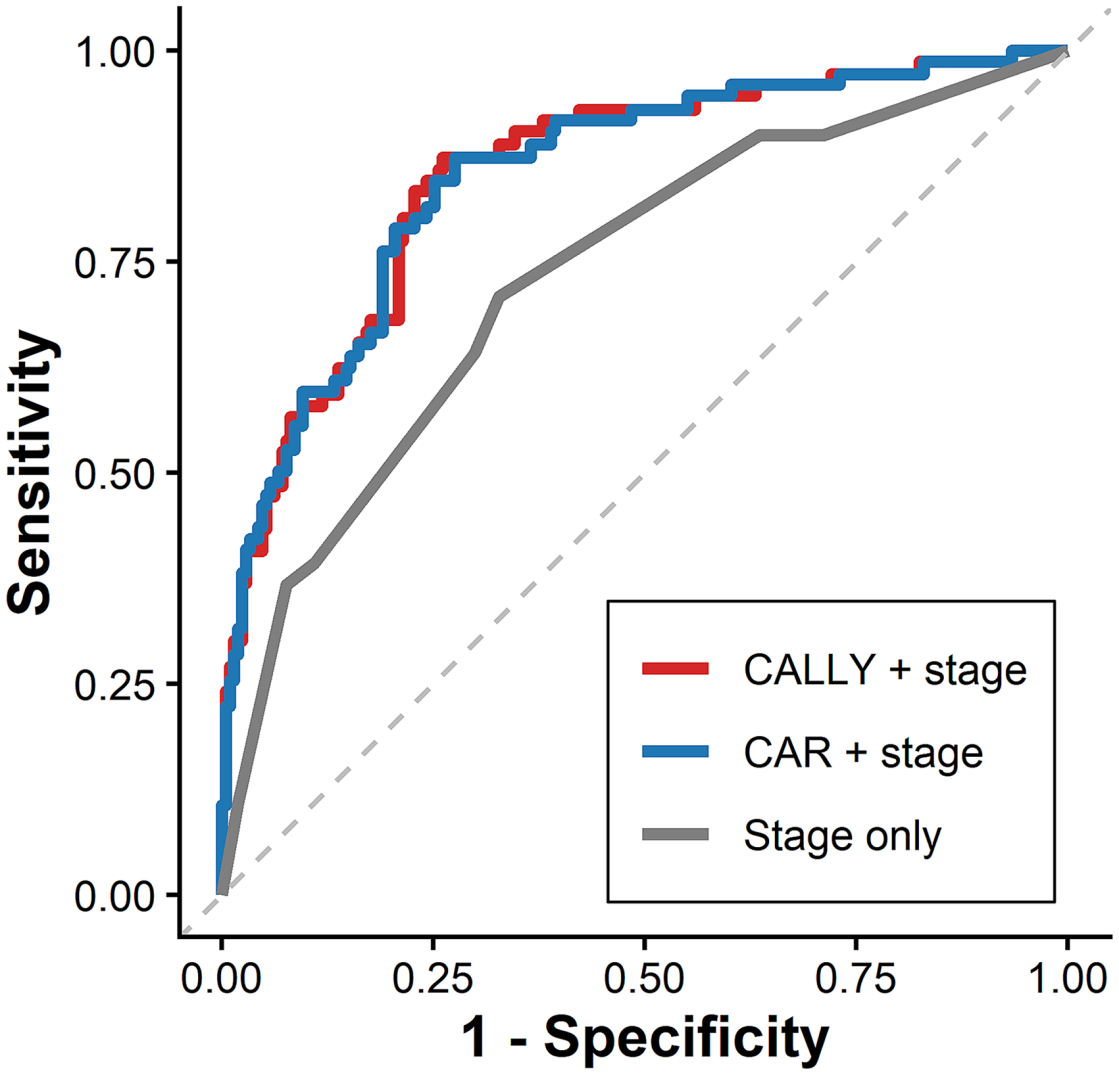
Time-dependent ROC curves of the stage-only model with and without the incorporation of the CALLY index or CAR for predicting 3-year DFS. The AUC for 3-year DFS was 0.735 (95% CI, 0.670–0.799) for the stage-only model. The AUC was 0.855 (95% CI, 0.818–0.912) for the CALLY + stage model and 0.854 (95% CI, 0.816–0.910) for the CAR + stage model. Compared with the stage-only model, the addition of the CALLY index resulted in an absolute AUC increase of 0.120 (*p* < 0.001), while the addition of CAR led to an absolute increase of 0.118 (*p* < 0.001). A pairwise comparison between the CALLY + stage and CAR + stage models revealed no significant difference in prognostic accuracy (absolute difference = 0.001; 95% CI, −0.005 to 0.015; *p* = 0.799). AUC, area under the curve; CAR, C-reactive protein-to-albumin ratio; CALLY, CRP-albumin-lymphocyte index; CI, confidence interval; DFS, disease-free survival; ROC, receiver operating characteristic.

The clinical utility of these markers was further demonstrated by their incremental prognostic value when incorporated into the T and N stages. The baseline stage-only model yielded a C-index of 0.713 (95% CI, 0.657–0.764). Adding the CALLY index increased the C-index to 0.812 (95% CI: 0.781–0.867), representing an absolute difference of 0.099 (95% CI, 0.063–0.163; *p* < 0.001). The CALLY+stage model also achieved an AUC of 0.855, with an absolute difference of 0.120 (*p* < 0.001) compared with the stage-only model in the 3-year DFS analysis (Figure 4).

Similarly, the CAR+stage model significantly enhanced prognostic accuracy, with a C-index of 0.809 (95% CI, 0.779–0.865; absolute difference, 0.097; *p* < 0.001) and a 3-year DFS AUC improvement (absolute difference, 0.118; *p* < 0.001) compared with the stage-only model. No significant difference in prognostic performance was observed between the CALLY-based and CAR-based integrated models (absolute difference, 0.003; *p* = 0.799).

## Discussion

This study evaluated the independent prognostic value of seven INMs in a cohort of 414 patients with nonmetastatic CRC. While all markers were significant in the univariable analysis, only the CALLY and CAR remained independent predictors of DFS after adjusting for covariates, including T and N stages. Integrating CALLY and CAR into the staging system significantly improved the predictive accuracy for DFS, with the C-index increasing from 0.713 to 0.812 and 0.809, respectively. These findings suggest that CALLY and CAR provide the robust and consistent independent prognostic information beyond conventional TNM staging in patients with nonmetastatic CRC.

A notable observation in this study was the discrepancy in the prognostic significance of lymphocyte-related markers between the screening phase and the multivariable models. In the initial evaluation (Supplementary Table S1), indices incorporating lymphocyte counts, such as PNI and ALI, showed higher predictive accuracy for DFS than other markers. However, after adjusting for T and N stages (Model 2), these markers lost their independent prognostic value. This finding suggests that the prognostic information provided by peripheral lymphocyte count may overlap with the tumor’s anatomical extent.

Biologically, lymphocytes are the fundamental effectors of host antitumor immunity, participating in the recognition and cytolytic destruction of malignant cells (20). While lymphopenia has been established as an independent risk factor for poor prognosis in CRC (21, 22), our findings imply that its intrinsic prognostic value may be diluted when incorporated into composite indices alongside other potent systemic indicators. This is supported by a direct comparison between the CALLY index and CAR. Despite including a lymphocyte component, the CALLY index did not yield a statistically significant improvement in overall predictive accuracy compared with CAR (C-index difference = 0.003; *p* = 0.799). Consequently, while lymphocyte-related markers showed high accuracy in univariable models, their independent contribution appeared attenuated in multivariable analysis, where they competed with more dominant predictors, including the pathological T and N categories.

The independent predictors in this study, CALLY and CAR, both incorporate CRP, which appears to be the most significant discriminatory factor among the evaluated indices. Postoperative CRP is a well-established acute-phase reactant used to detect short-term complications (23), and recent evidence suggests that both preoperative and postoperative CRP levels are significant predictors of long-term survival (24). Our findings provide indirect evidence that preoperative CRP levels have unique prognostic value, distinct from that provided by T and N stages.

Several biological pathways mediate the involvement of CRP in the long-term prognosis of CRC. Although CRP is a nonspecific acute-phase reactant, its synthesis in cancer patients is thought to be largely driven by tumor-derived pro-inflammatory cytokines, such as interleukin-6 (IL-6) and tumor necrosis factor-alpha (TNF-*α*) (25, 26). The resulting systemic inflammatory response (SIR) has been proposed to promote tumor proliferation, angiogenesis, epithelial-mesenchymal transition (EMT), and the establishment of pre-metastatic niches (27, 28). Another potential mechanism involves CRP’s effect on the host immune response: elevated CRP levels have been associated with depletion of peripherally circulating lymphocytes (29). The biological interpretations presented above were inferred from established findings in prior studies rather than directly demonstrated in our cohort, and warrant confirmation through future translational research.

Despite the inclusion of CRP, mGPS failed to maintain independent prognostic significance in our T and N-adjusted multivariable model (Model 2). One possible explanation is that the fixed threshold of the mGPS (> 1.0 mg/dL) is insufficient to capture subtle prognostic variations in non-metastatic CRC. To better understand why mGPS lost significance despite incorporating CRP, we conducted a post-hoc restricted cubic spline (RCS) analysis to characterize the functional relationship between CRP and DFS (Supplementary Figure S2). The HR for DFS began to rise at CRP levels around 0.1–0.2 mg/dL, and by the time CRP reached 1.0 mg/dL, the risk of recurrence had already increased substantially. These data suggest that the mGPS primarily identifies patients whose risk has already escalated. Leveraging the continuous nature of these parameters, CALLY and CAR allow for more refined risk stratification than the categorical mGPS scoring system in this patient population.

The incremental prognostic value observed in this study (C-index increase: 0.097) represents a substantial improvement in predictive accuracy. In the existing literature on prognostic models for CRC, the addition of a single biomarker or clinicopathological factor typically yields a C-index increase ranging from 0.02 to 0.06. Gao and colleagues (30) observed an OS improvement of 0.062 (0.735 to 0.797) when LVI was added in stage II patients. Similarly, Hu et al. (31) recently analyzed DFS in a stage I–III cohort and reported a gain of 0.061 (0.741–0.802) after incorporating both LVI and PNI into the TNM system.

These differences may be attributed to methodological and biological factors. First, while traditional pathological markers such as LVI and PNI are categorical, the CALLY index and CAR are continuous variables that capture subtle physiological variations. Furthermore, the use of DFS as the primary outcome may have enhanced sensitivity, as systemic inflammation, immunosuppression, and malnutrition are more directly linked to recurrence than to overall mortality. Although these findings emphasize the clinical utility of CALLY and CAR, other unmeasured factors, such as specific molecular subtypes or post-treatment inflammatory shifts, may also influence outcomes. Future research integrating molecular markers and longitudinal changes in systemic inflammation could further validate and refine these results across diverse patient populations.

The identification of CALLY and CAR as independent prognostic markers has implications for perioperative nutritional management. Serum albumin alone is a nonspecific nutritional indicator, as hypoalbuminemia can arise from diverse etiologies unrelated to tumor biology. By incorporating CRP, CALLY and CAR can distinguish patients whose nutritional deterioration is driven by tumor-associated systemic inflammation—a dimension not directly captured by current screening tools such as the Nutritional Risk Screening 2002 and Subjective Global Assessment (32). In an exploratory subgroup analysis, low CALLY and high CAR were associated with markedly poorer outcomes even among stage II patients who did not receive adjuvant chemotherapy (Figure 2), raising the possibility that these markers could help identify candidates who might benefit from adjuvant treatment or closer surveillance. However, given the small subgroup size and univariable nature of this analysis, this finding should be interpreted as a preliminary observation rather than a definitive conclusion. Further multicenter studies with external validation are needed to establish these markers as robust prognostic indicators before their integration into clinical decision-making can be considered.

This study has several limitations to consider when interpreting the results. First, its retrospective, single-center design may have introduced unmeasured confounding factors and limited the generalizability of our findings. As INMs are sensitive to institutional laboratory standards, perioperative care, and patient demographics, the proposed cutoffs and model performance may not be directly reproducible in other settings, and external validation in independent, preferably multicenter cohorts is therefore warranted. Second, although the median follow-up period of 36.3 months was sufficient to capture most recurrent events in nonmetastatic CRC, longer-term observation is necessary to evaluate the prognostic impact of these markers fully. Third, the use of pathologic staging rather than clinical staging could be debated, particularly in patients with rectal cancer. Approximately one-third (57/170) of patients with rectal cancer received neoadjuvant chemotherapy or radiotherapy, which could have led to the downstaging of their pathologic results. However, because the cohort also included patients with colon cancer for whom CT scans do not provide accurate clinical T and N staging, we prioritized consistency by using pathologic staging across the entire study population. Fourth, several molecular and pathological variables were not incorporated into the primary multivariable models owing to constraints on model parameters and high rates of missing data. Among the molecular markers, MSI/MMR and *KRAS* status—which had relatively low missing rates—were additionally examined in a sensitivity analysis using the same multiple imputation approach, and both the CALLY index and CAR retained independent prognostic significance after their inclusion (Supplementary Table S2). However, *BRAF* mutation status, tumor budding, extranodal extension, and detailed information on adjuvant chemotherapy (including reasons for its omission) could not be incorporated owing to high rates of missing data or limited availability, and their potential confounding effects cannot be excluded. Finally, our analysis was restricted to single-point preoperative measurements and did not account for longitudinal fluctuations in inflammatory or nutritional status that may occur postoperatively or during adjuvant therapy. Future studies incorporating dynamic changes in these markers may further enhance the precision of CRC prognostic models.

In conclusion, among the seven evaluated IMNs, the CALLY index and CAR were identified as independent prognostic factors for nonmetastatic CRC. When combined with TNM staging, these markers improved DFS predictive accuracy, effectively addressing the limitations of the current staging system.

## Data Availability

The raw data supporting the conclusions of this article will be made available by the authors, without undue reservation.
