# Supplementary material for: Which immunonutritional marker best predicts disease-free survival in non-metastatic colorectal Cancer? The CALLY index and CAR emerge as independent predictors from a seven-marker comparison
Source: Front Nutr. 2026 Jun 18;13:1865242. doi: 10.3389/fnut.2026.1865242 (PMC13323131; doi:10.3389/fnut.2026.1865242)
Supplement: Supplementary file 1 [file Supplementary_file_1.docx]

| **Supplementary Table S1. C-index of candidate immuno-nutritional markers for predicting disease-free survival.** | |
| --- | --- |
| Markers | C-index (95% CI) |
| ALI | 0.651 (0.587–0.715) |
| HALP | 0.601 (0.542–0.660) |
| CALLY | 0.649 (0.588–0.710) |
| CAR | 0.624 (0.558–0.689) |
| PNI | 0.663 (0.603–0.723) |
| CONUT | 0.618 (0.560–0.675) |
| mGPS | 0.560 (0.510–0.610) |
| Abbreviations: ALI, advanced lung cancer inflammation index; HALP, hemoglobin, albumin, lymphocyte, and platelet; CALLY, CRP-albumin-lymphocyte; CAR, C-reactive protein-to-albumin ratio; PNI, prognostic nutritional index; CONUT, controlling nutritional status; mGPS, modified Glasgow Prognostic Score; CI, confidence interval; DFS, disease-free survival. | |

**Supplementary Figure S1. Flow chart of patient inclusion.**


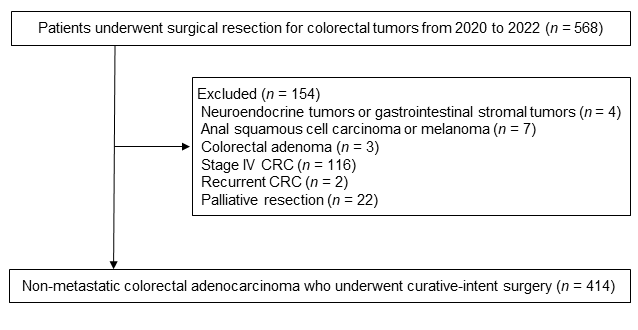


**Supplementary Figure S2. Restricted cubic spline analysis of the dose-response relationship between serum C-reactive protein and the risk of recurrence.**


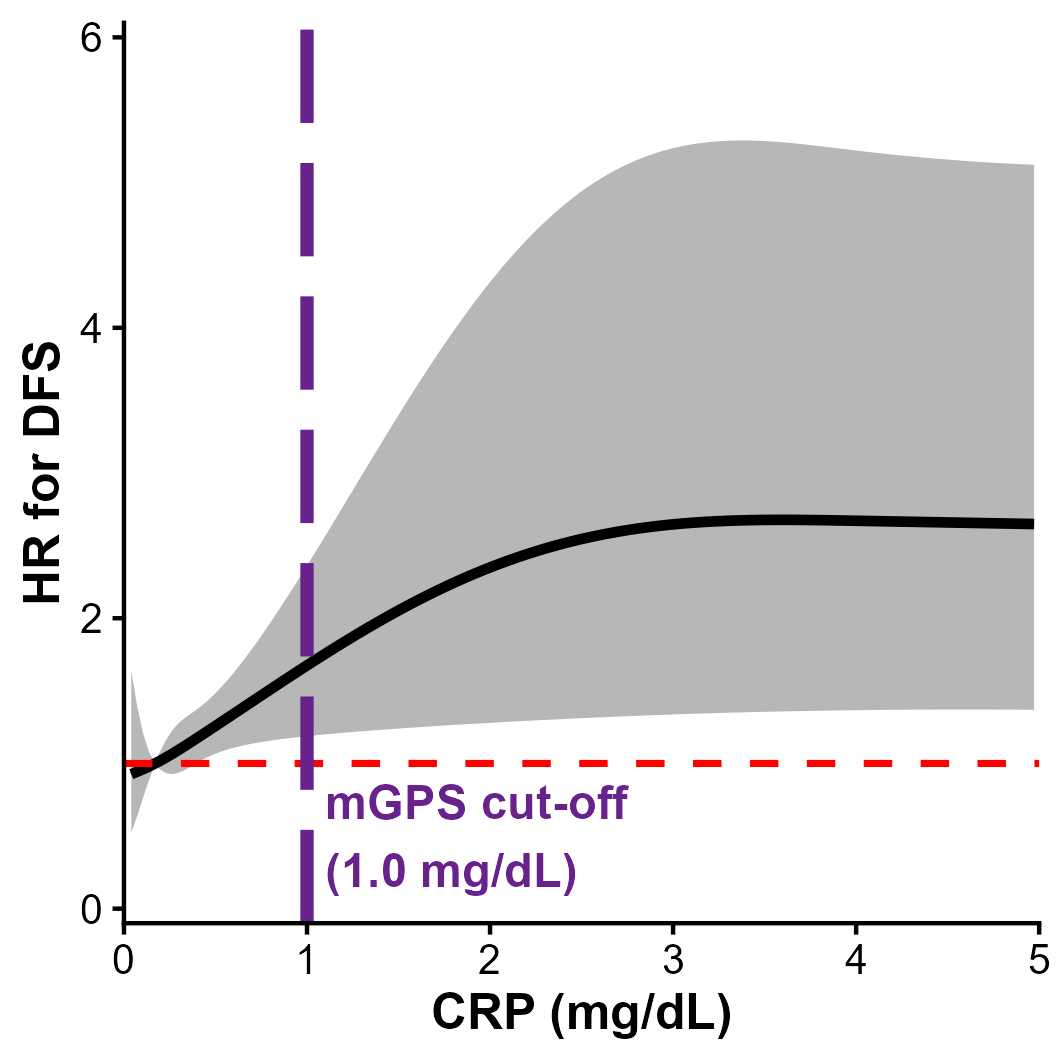


| **Supplementary Table S2. Multivariable Cox proportional hazards models for disease-free survival incorporating MSI- and KRAS mutation status.** | | | | | |
| --- | --- | --- | --- | --- | --- |
|  | CALLY model | |  | CAR model | |
|  | HR (95% CI) | *P* |  | HR (95% CI) | *P* |
| CALLY < 1.43 | 1.852 (1.133-3.026) | 0.014 |  | - | - |
| CAR > 0.13 | - | - |  | 2.247 (1.363-3.706) | 0.002 |
| Age >70 years | 2.449 (1.436-4.177) | 0.001 |  | 2.337 (1.375-3.974) | 0.002 |
| ASA PS ≥ III | 1.360 (0.783-2.363) | 0.270 |  | 1.397 (0.808-2.413) | 0.227 |
| CEA >5 ng/ml | 1.654 (1.000-2.734) | 0.049 |  | 1.670 (1.012-2.756) | 0.045 |
| Neoadjuvant Tx. | 5.745 (3.054-10.805) | < 0.001 |  | 6.397 (3.380-12.106) | < 0.001 |
| T stage |  |  |  |  |  |
| 1-2 | Ref |  |  | Ref |  |
| 3 | 4.096 (1.513-11.091) | 0.006 |  | 4.083 (1.503-11.093) | 0.006 |
| 4 | 6.552 (2.041-21.036) | 0.002 |  | 6.630 (2.065-21.289) | 0.001 |
| N stage |  |  |  |  |  |
| 0 | Ref |  |  | Ref |  |
| 1 | 1.785 (0.936-3.402) | 0.077 |  | 1.804 (0.953-3.416) | 0.069 |
| 2 | 4.729 (2.360-9.479) | < 0.001 |  | 4.553 (2.297-9.025) | < 0.001 |
| LVI | 0.590 (0.294-1.184) | 0.134 |  | 0.587 (0.296-1.164) | 0.124 |
| NI | 1.512 (0.770-2.969) | 0.225 |  | 1.476 (0.756-2.882) | 0.249 |
| R1 resection | 1.533 (0.790-3.052) | 0.197 |  | 1.469 (0.744-2.899) | 0.262 |
| Adjuvant CTx | 0.732 (0.372-1.439) | 0.359 |  | 0.734 (0.374-1.441) | 0.362 |
| MSI-H | 0.822 (0.240-2.815) | 0.751 |  | 0.821 (0.238-2.827) | 0.750 |
| *KRAS* mutation | 1.461 (0.899-2.374) | 0.123 |  | 1.454 (0.898-2.354) | 0.125 |
| Each model was adjusted for individual pathologic T and N stages and other covariates, with MSI/MMR and *KRAS* status additionally included. Missing values were handled using multiple imputation (m = 20), consistent with the primary analysis.  Abbreviations: HR, hazard ratio; CI, confidence interval; ASA PS, American Society of Anesthesiologists physical status; CEA, carcinoembryonic antigen; Tx, therapy; LVI, lymphovascular invasion; NI, neural invasion; R1, microscopic residual tumor; CTx, chemotherapy; MSI-H, high microsatellite instability; CALLY, CRP-albumin-lymphocyte index; CAR, C-reactive protein-to-albumin ratio; Ref, reference category. | | | | | |
